# Practicing pharmacist education based on the experiences of medical support on the cruise ship Diamond Princess

**DOI:** 10.1186/s12909-025-07291-8

**Published:** 2025-05-16

**Authors:** Yoshio Kusakabe, Yasuhiro Nakamura, Keiji Maruyama

**Affiliations:** Faculty of Pharmaceutical Sciences, Teikyo University, 2-11-1 Kaga, Itabashi-ku, Tokyo, 173-8605 Japan

**Keywords:** Cruise ship Diamond Princess, Education, Pharmacy, Professionalism, AI

## Abstract

**Background:**

Passengers on the cruise ship Diamond Princess (DP), which departed Yokohama on 20 January 2020, were found to be infected with the new coronavirus after arrival in Hong Kong. Passengers and crew were not allowed to disembark, instead being quarantined on board; an onboard pandemic resulted. Many passengers were elderly and in need of medications; pharmacists and other professionals, including the author, were assigned to provide medical support (the author participated on two occasions). Many passengers were not Japanese nationals; those who required medicines not sold in Japan received analogs of medicines that are sold in Japan. Pharmacists were required to complete medication guidance documents (in English). The author considered that by using this experience as teaching material, pharmacy students would not only learn English but also become educated in terms of drug therapy.

**Method:**

The author created an exercise for second-year students at Teikyo University in which they were required to provide real-world medical support. The educational effects were measured by analyzing the answers to questionnaires completed before and after the exercise and ‘Impressions of the exercise’ homework.

**Results:**

Using real emergency events as a teaching tool enhanced students’ motivation to learn English and pursue professional pharmacy education (the latter was scheduled to begin in earnest in the third year). At that time, the new coronavirus was poorly understood. The author’s experiences taught students that medical workers are educated to offer care even when they are at risk of infection. Translation software (a form of artificial intelligence [AI]) was used to create medication guidance documents in English. The students learnt that if AI translations, i.e., medication guidance documents in English, were accepted at face value, they would be held responsible if the documents were in error.

**Conclusion:**

By both listening to the author’s lecture on a real-world medical support situation and completing an assignment, students learned many things that are difficult to teach via lectures alone, including the dangers arising when using AI technology in clinical settings and the mindset of medical professionals.

## Background

Coronavirus 2019 (COVID-19) infection is caused by severe acute respiratory syndrome-related coronavirus 2; it was initially reported in China in December 2019 and remains prevalent worldwide [1–4]. On 30 January 2020, the World Health Organization formally declared this new coronavirus a public health emergency of international concern [5]. The Diamond Princess (DP) cruise ship departed Yokohama on 20 January 2020 and was scheduled to cruise for about 2 weeks, visiting Southeast Asia and then returning to Yokohama. However, during the voyage, one passenger who disembarked in Hong Kong was found to be infected with COVID-19 [6]. When the DP stopped at Yokohama COVID-19 polymerase chain reaction tests were performed on all crew and passengers. Some of the test results were positive, and no passengers were allowed to disembark [7].

At the time, there was no effective treatment or vaccine for COVID-19, and it was not clear how Japan would deal with the unknown virus. The world watched on because the cruise ship hosted > 3700 people from 56 countries. The DP was registered abroad, so the medical treatments available on board differed from those in Japan. Japanese pharmacists provided medical support from an off-board quarantine station. Many medicines sold in Japan were stocked in this off-board quarantine station, and the dispatch pharmacists sought to provide those medicines on request. However, the DP also had many non-Japanese passengers, who made many requests for medicines not sold in Japan. In such cases, an alternative drug was selected from among domestically available drugs at the discretion of a pharmacist. Direct pharmacist-patient interaction was not permitted, so medication instructions and precautions were written on paper in English and delivered to passengers in sealed plastic bags by the Japan Self-Defense Forces [8–10].

In February 2020, the first author (Yoshio Kusakabe, Ph.D.) participated in these medical support efforts as a pharmacist. This experience with the DP revealed the challenges pharmacists face when providing medical support in multilingual disaster settings. In particular, effective communication in English and the ability to assess artificial intelligence (AI)-generated translations are critical skills. AI translation tools have become widely available [11], but their use in clinical settings poses potential risks, as mistranslation of drug instructions could lead to serious medical errors [12, 13]. Therefore, pharmacists must critically evaluate AI-generated content rather than relying on it unconditionally [14, 15].

Despite the growing importance of AI literacy in healthcare, pharmacy education in Japan does not systematically address AI-related risks. At the Faculty of Pharmaceutical Sciences at Teikyo University, for example, AI is only briefly introduced in a first-year course on information literacy, which primarily focuses on general internet usage. No specialized coursework has been developed to educate future pharmacists on AI-related challenges in clinical practice.

To address these gaps, a seminar was developed for second-year pharmacy students as part of a required course (Medical Communication 1). The seminar incorporated real-world examples from the DP disaster response to improve student awareness of the importance of English proficiency in pharmaceutical practice, the ethical and practical implications of AI use in healthcare, and the professionalism required in emergency medical situations. Unlike traditional lectures, this seminar was developed to provide students with hands-on learning opportunities, simulating real-world scenarios in which pharmacists must navigate language barriers and AI limitations as they engage in clinical decision-making. This article explores changes in awareness of these key competencies before and after the seminar.

Pharmacy education in Japan involves a 6-year program, culminating in a national pharmacist licensure examination. Students typically spend the first 2 years studying basic liberal arts, biology, and chemistry before transitioning to more clinically focused coursework. Some students struggle to connect these foundational subjects with pharmacotherapy practice, which may decrease their motivation to engage with them.

Medical Communication 1 is a compulsory second-year course of the Faculty of Pharmaceutical Sciences at Teikyo University. It is intended to promote ethical awareness, communication skills, and professional identity among pharmacy students. The seminar on the DP disaster was developed to bridge the gap between early-stage pharmaceutical education and clinical practice. By focusing on a real-world disaster case study, the goal was to enhance student motivation to learn English, deepen their understanding of AI-related risks, and reinforce the importance of professionalism in healthcare.

To date, no previous reports have leveraged pharmacist-led medical support experiences on the DP as an educational tool in pharmacy training. Given the increasing emphasis on professionalism in medical education, integration of this kind of disaster-related case study into pharmacy curricula may contribute to more effective professional development.

The participants in this study were second-year pharmacy students who had completed some foundational coursework in liberal arts, biology, and chemistry. At the time of the study, they were beginning to apply their theoretical knowledge to clinical scenarios. The real-world scenario of the DP was intended to give students a more comprehensive understanding of the role of pharmacists in disaster situations, and specifically the necessity of English proficiency in medical communication, and the ethical considerations involved in the use of AI-assisted translation tools.

## Methods

### Overview of the exercise

Participating students completed a pre-questionnaire before starting the seminar. The seminar began with a lecture detailing the experience of providing medical support onboard the DP in February 2020. It emphasized the challenges faced, including working in a high-risk COVID-19 environment with limited information, and the essential role of pharmacists in a disaster response. It also highlighted the risks of AI software, particularly in generating medication instructions in English, and reinforced the pharmacist’s responsibility in ensuring accuracy.

After the lecture, students were assigned a task: in response to requests from three passengers, provide alternative Japanese drugs and accompanying instructions in English to ensure the drug is taken safely. The requested medications were Aprovel (150 mg; half a tablet per day), Robitussin Cough and Chest Congestion DM (118 mL), and BreathRX (an anti-bacterial tongue spray for dental implants).

None of these medications are sold in Japan, so the students needed to determine appropriate alternative medications that could be safely used by the passengers. This task revealed the need for pharmacists not only to possess pharmacological knowledge, but also to have effective communication skills so that they can provide appropriate medication guidance in English.

Students worked in small groups to select appropriate alternative medications, but were required to individually create and submit medication guidance texts in English. As homework, the students were assigned an essay (minimum of 500 words) reflecting on their experiences during the seminar. At the conclusion of the lecture, students were told that if they did not want to participate in the study, they could opt out by contacting the authors. No students opted out.

The seminar was conducted in a classroom setting, enabling face-to-face direct interaction between students and instructors. The lecture, group discussions, and task-based activities all took place in person so students could engage in real-time discussions and receive immediate feedback. No individuals were present during the seminar other than the participants and researchers; the lecture and group activities were conducted solely by the research team, and no external observers, educators, or assistants were involved.

### Participants and sampling

The seminar was integrated into the second-year curriculum of the Faculty of Pharmaceutical Sciences at Teikyo University as part of the Medical Communication 1 course on 23 June 2023, and 334 students participated. The male-to-female ratio was approximately 38:62, and 98% of the participants were aged 19–21 years. Medical Communication 1 is a mandatory course, so no *a priori* selection criteria were applied.

The first author has a background in pharmaceutical education, ethics, and medical communication, and has conducted three lectures for the Medical Communication 1 course before this seminar, ensuring familiarity with the course objectives and student expectations. None of the researchers had any prior personal or academic relationships with students, minimizing the potential for bias.

### Educational context and course description

In Japan, the 6-year pharmacy program culminates in a professional degree (PharmD equivalent). During the first and second years, the curriculum primarily focuses on liberal arts and basic sciences, making it difficult for students to connect their studies with clinical practice, and some students have minimal enthusiasm for pharmaceutical education. Although the need to foster professionalism among pharmacists is being increasingly emphasized, systematic approaches focusing on this aspect remain limited.

This seminar was developed to address these challenges, drawing from the real-world medical support scenario of the DP outbreak to help students understand the relevance of their studies to clinical practice. Some elements of medical experiences related to the DP disaster have been incorporated into nursing curricula, but this is the first report of a similar seminar in the context of pharmacy education. The seminar provided opportunities for pharmacy students to engage in a simulated medical support scenario that emphasized the importance of English proficiency, AI-related risks in clinical settings, and the role of pharmacists in disaster response.

The seminar required students to select alternative Japanese medications for foreign patients and explain their use in English, encouraging students to develop their pharmaceutical knowledge, communication skills, and awareness of the professional responsibilities of pharmacists. Given the increasing use of AI-based translation tools in healthcare, it was also designed to raise awareness of the potential risks and limitations associated with AI-assisted communication in clinical practice. The goals were to enhance student motivation for English learning, deepen their understanding of pharmaceutical care, and foster a stronger sense of professionalism in both routine and emergency medical settings [16].

### Questionnaire design and validation

**Fig. 1:**
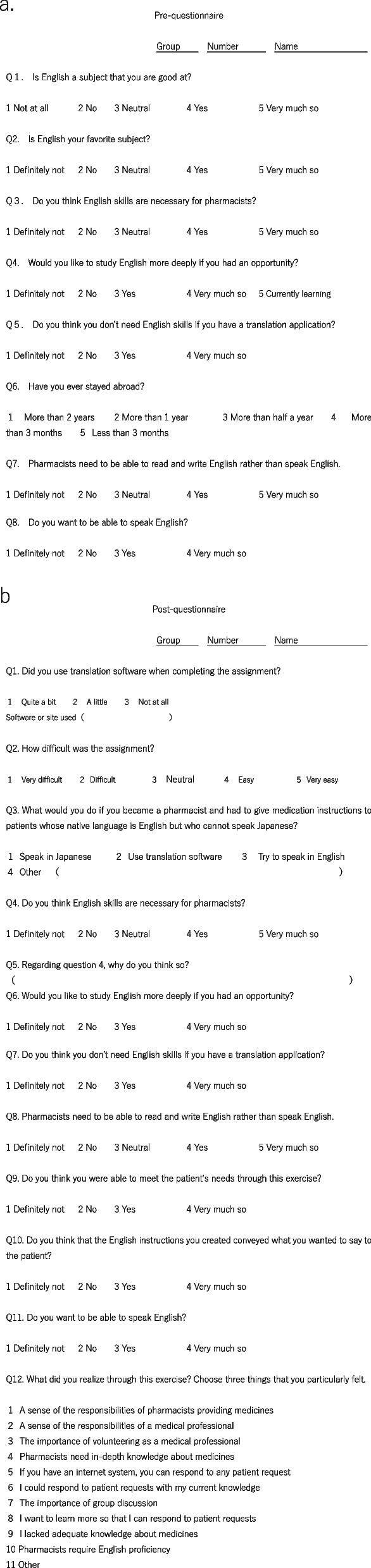
Pre- (**a**) and post- (**b**) questionnaires

A pre-questionnaire (Fig. 1a) was administered before the seminar, and a post-questionnaire (Fig. 1b) was completed afterward. The post-questionnaire included the same questions as those of the pre-questionnaire, investigating changes in student perceptions of the importance of English language proficiency, attitudes about the medical profession, and perceptions of AI-related risks in clinical practice.

As noted above, the first- and second-year curricula at Japanese pharmacy schools include many basic liberal arts, biology, and chemical courses, and some students find it difficult to understand how some of these subjects relate to drug treatment, which can reduce their motivation to study. The need for professionalism among medical professionals is also increasingly being emphasized. Overall, systematic learning approaches are needed, but to date no reports have documented pharmacist education programs that are designed to foster professionalism. Our seminar conveyed the realities of medical settings during actual disasters and encouraged students to engage with the scenarios: this compulsory education was intended to enhance motivation among second-year students to learn more about AI, professionalism, and communication related to drug treatment.

The questionnaire was designed to assess student perceptions about English proficiency, attitudes about AI, and professionalism in pharmaceutical practice. All questions, as well as instructional prompts and guidelines, were developed by the authors in alignment with the educational objectives of the seminar. Questions 1 and 2 pertained to the liking of and proficiency in English, and were included to determine whether difficulty in completing the assignment was due to language barriers or limited pharmacological knowledge. Questions also explored whether the motivation of students who recognized the importance of English proficiency in the context of pharmacy was affected by their language ability or initial struggles with the task.

Given the increasing reliance on AI-assisted translation tools, the questionnaire also investigated whether students who were less confident in English were more likely to depend on AI and whether they were aware of AI-related risks in clinical practice.

To ensure the reliability of the findings, identical pre- and post-questionnaire questions were used for comparative analysis. One pre-questionnaire item (“Would you like to study English more deeply if you had an opportunity?”) had an additional response option (“Currently learning”), and students selecting this option were excluded from the analysis. No formal pilot test was conducted, but the questions were reviewed by experienced faculty members working in the field of pharmaceutical education to ensure validity.

### Data collection methods

Data were collected from the pre- and post-questionnaires, group discussions, and qualitative content analysis of the students’ homework. Post-questionnaire measured changes in perceptions of English communication, use of AI, and professionalism after the seminar. To ensure consistency in the data collection process, pre- and post-questionnaires  responses were collected in the classroom. No audio or visual recordings were made during the study, and students were assured that their responses would be anonymized and used for research purposes only. Homework assignments were submitted individually online and analyzed qualitatively. No interviews or focus groups were conducted, so no transcripts were returned to the participants for comment or correction.

### Statistical analysis

Responses to the pre- and post-questionnaires were analyzed using GraphPad Prism (ver. 9.4.1; GraphPad Software, Inc., San Diego, CA, USA) and Microsoft Excel (Microsoft Corp., Redmond, WA, USA) software. Responses on identical pre- and post-questionnaire questions were statistically compared to determine changes in student awareness (Pre-Q3 vs. Post-Q4, Pre-Q5 vs. Post-Q7, Pre-Q4 vs. Post-Q6, Pre-Q7 vs. Post-Q8). The Wilcoxon signed-rank test, a non-parametric test suitable for analyzing paired data, was performed to detect significant changes because of its ability to assess differences in ordinal data without the assumption of a normal distribution, making it appropriate for evaluating changes in questionnaire responses.

### Qualitative content analysis

Qualitative data were independently coded by two researchers to ensure reliability [17, 18]. Any discrepancies in coding were discussed and resolved. Homework assignments reflecting on “Impressions of the seminar” were subjected to qualitative content analysis to explore perceptions and learning outcomes. All names and identifying information were removed before analyses to ensure anonymity. Two analytical approaches were employed, as follows.

#### Text mining and morphological analysis

KH Coder text analysis software was used to conduct morphological analysis and generate a co-occurrence network diagram. First, all student assignments (*N* = 291) were tokenized using MeCab, a Japanese morphological analysis software package [19, 20]. The 10 most frequently occurring nouns were then extracted from the students’ reflections and visualized to identify key themes and relationships. A co-occurrence network diagram was created to illustrate the relationships among frequently mentioned words. The diagram was initially generated in Japanese and later translated into English.

#### Qualitative thematic analysis

A manual coding process was used for thematic coding and categorization, utilizing MAXQDA Analytics Pro 2022 software to identify recurring themes in the students’ reflections [21, 22]. Responses were categorized into key themes such as the need for English proficiency, AI risks, medical professionalism, and the application of pharmaceutical knowledge in emergency scenarios. Two researchers independently coded the responses to enhance reliability, and any discrepancies were resolved through discussion.

#### Coding framework

Themes were derived inductively from student responses using an open coding approach. Two researchers independently performed the initial coding and then grouped recurring patterns into categories. A structured coding tree was developed through an iterative process, and the final coding framework was refined through discussion to ensure consistency and reliability. The framework consisted of three main themes with subcategories as follows: importance of English proficiency (motivation for learning English; challenges in using English in pharmacy practice; perceived impact of English proficiency on professional competence), awareness of AI risks in clinical practice (perceived reliability of AI-based translation tools; concerns about misinterpretation of medication instructions; trust in human verification over AI-generated content), and professionalism in pharmacy practice (recognition of pharmacists’ roles in disaster response; ethical considerations in pharmaceutical communication; awareness of responsibilities in patient education).

The final coding tree was reviewed by both researchers to ensure inter-rater reliability.

By integrating text mining and qualitative coding, a comprehensive understanding of student feedback was obtained, allowing for objective evaluation of key themes and trends. Representative quotes from students were selected to illustrate key themes, ensuring that their voices were adequately reflected in the results. The primary facilitator reflected on any potential biases and preconceptions to mitigate their impact on the interpretation of the study data.

A sufficient number of homework assignments (*N* = 291) was analyzed to ensure a diverse range of perspectives was captured. Thematic saturation was considered to have been reached when no new themes emerged during qualitative coding.

In addition to the primary themes identified, some students expressed unique perspectives that did not fit neatly within the main categories. For example, a few students reported concerns about overreliance on AI translation tools in clinical settings, fearing that these might discourage pharmacists from developing their own English communication skills. Others mentioned the psychological burden of providing recommendations about medication in a foreign language, highlighting the need for more emotional support and training in pharmacy education. These comments were less frequent, but still provide valuable insights into individual differences in learning experiences. Based on these findings, future iterations of the course could incorporate more targeted interventions, such as confidence-building seminars for English communication and discussions of ethical concerns regarding AI use in pharmacy practice.

### Ethical considerations

This research adhered to the Ethical Guidelines for Life Science and Medical Research Involving Human Subjects and the Declaration of Helsinki. It was approved by the Teikyo University Ethical Review Board for Medical and Health Research Involving Human Subjects (approval no. 23–131). Students were required to take the seminar as part of their mandatory course, but were informed that their responses would not affect their grades. Students were informed that completion of the questionnaire was taken to indicate that they agreed to participate in the study, and that if they did not want to participate in the study, they could opt out by contacting the authors. No students opted out.

Participants did not provide feedback on the findings: no interviews or focus groups were conducted, so participants had no opportunities to review or comment on the results.

This study was not a clinical trial, so it does not have a clinical trial registration number.

## Results

### Questionnaire results

**Fig. 2:**
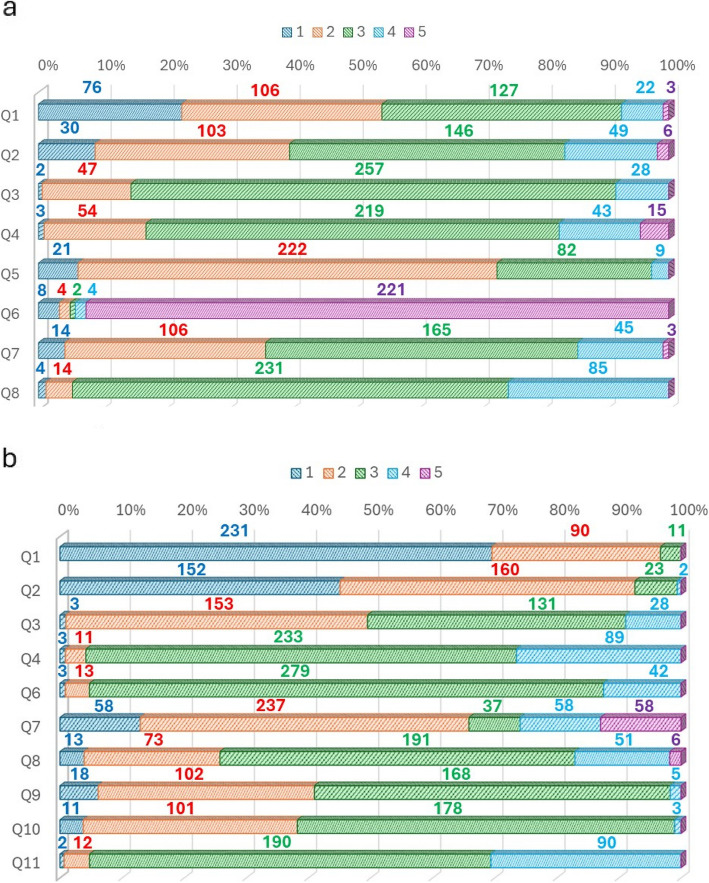
Aggregated responses to all questions on the pre-questionnaire (**a**) and post-questionnaire (**b**)

**Fig. 3:**
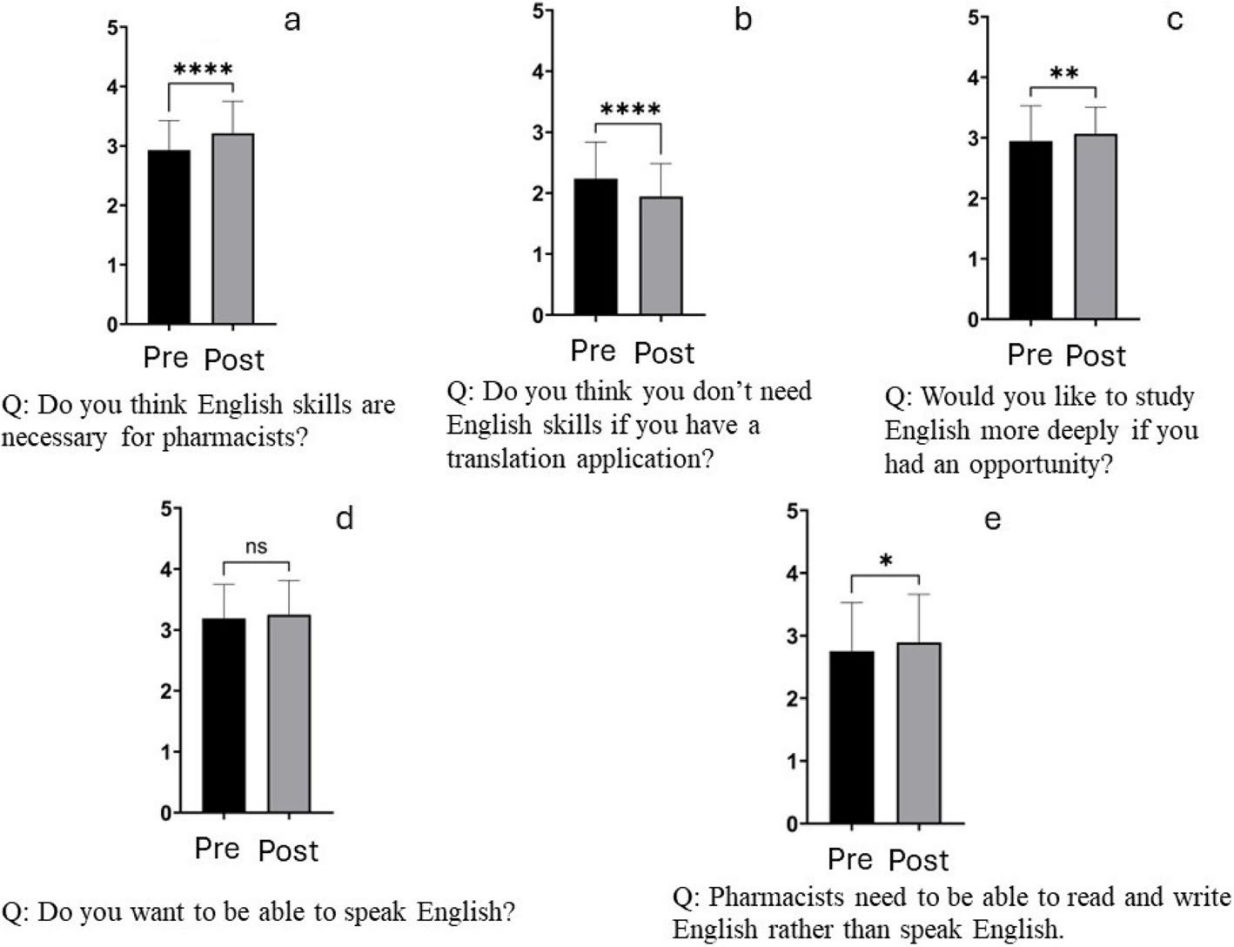
Responses to highly similar questions on the pre- and post-questionnaires. **a** Comparison before and after the exercise of the responses to the question ‘Do you think English skills are necessary for pharmacists?’. **b** Comparison before and after the exercise of the responses to the question ‘Do you think you don’t need English skills if you have a translation application?’. **c** Comparison before and after the exercise of the responses to the question ‘Would you like to study English more deeply if you have an opportunity?’. **d** Comparison before and after the exercise of the responses to the question ‘Do you want to be able to speak English?’ **e** Comparison before and after the exercise of the responses to the statement ‘Pharmacists need to be able to read and write English rather than speak English’

The results of the pre- and post-questionnaires are summarized in Fig. 2. Although the results of many questions on the pre- and post-questionnaires were the same, significant changes in the results were made to the questions ‘Do you think English skills are necessary for pharmacists?’ and ‘Do you think you do not need English skills if you have  a translation application?’ (Fig. 3a, b and Table 1). Specifically, the wording and context of these questions were adjusted to better capture the students’ perceptions and attitudes towards the importance of English skills in the pharmaceutical field and the reliance on translation applications. Furthermore, a significant change was made to the question ‘Would you like to study English more deeply if you had an opportunity?’ (Fig. 3c and Table 1). However, there was no significant change to the question ‘Do you want to be able to speak English?’ (Fig. 3d and Table 1). The number of ‘Yes’ responses to the statement ‘Pharmacists need to be able to read and write English rather than speak English’ increased significantly after the exercise (Fig. 3e and Table 1). This reflects a shift in students’ understanding of the practical application of English skills in their professional field (Table 2).

**Table 1:** Statistics of answers the highly similar questions on the pre- and post-questionnaire

Question	Pre-		Post-		*P* value
	Avg.	S.D.	Avg.	S.D.	
Do you think English skills are necessary for pharmacists?	2.931	0.495	3.214	0.537	<0.0001
Do you think you don’t need English skills if you have a translation application?	2.237	0.601	1.943	0.542	<0.0001
Would you like to study English more deeply if you had an opportunity?	2.947	0.583	3.068	0.442	0.002
Do you want to be able to speak English?	3.189	0.557	3.252	0.558	0.1575
Pharmacists need to be able to read and write English rather than speak English.	2.754	0.775	2.892	0.7674	0.0213

**Table 2:** The results of coding using MAXQDA for frequently occurring contents in ‘impressions of the exercise’ assigned as homework

Contents	Frequency
Pharmacists need English skills	277/291 95.2%
The author conveyed the reality of the experience by lecturing using photographs, and that students were able to perform their assignments with an awareness of the actual situation	128/291 44.0%
The need to make their own decisions rather than relying solely on answers provided by AI features such as translation apps	115/291 39.5%
The excellent drug knowledge, and their motivation to study drug treatment thus increased	67/291 23.0%
The significance of supplying medical support during disasters as medical professionals	90/291 30.9%
Increased interest in participating in such projects in the future	27/291 9.3%

**Fig. 4:**
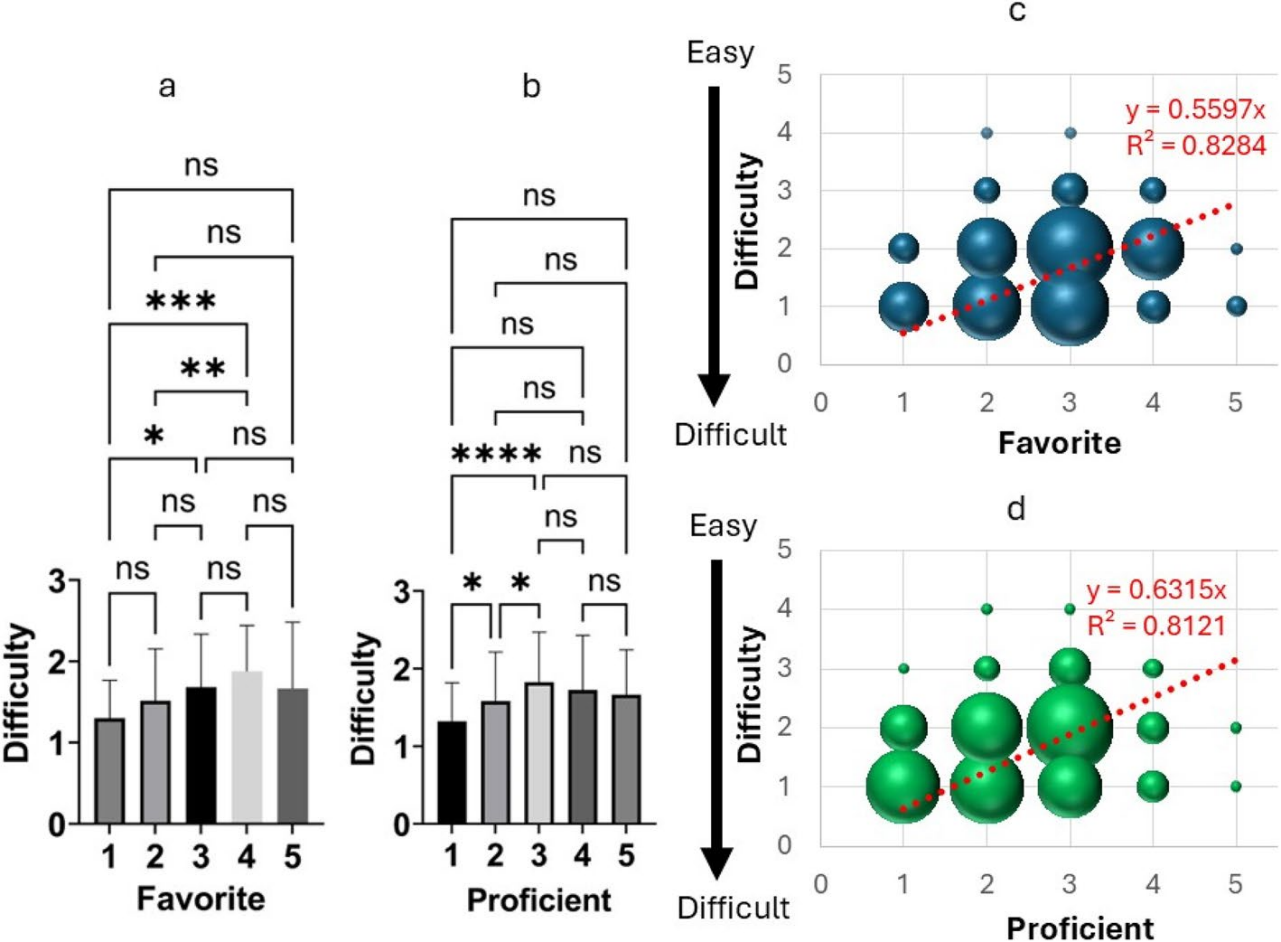
Relationship between the answers to the question ‘How difficult was the assignment?’ and familiarity with English. **a** Task difficulty ratings of students who did and did not like English. Students who liked and disliked English indicated how difficult they found the task. n.s., *p* ≥ 0.05; *p < 0.05; **p < 0.01; ***p < 0.001. **b** Relationship between task difficulty and English skills. Students with good and bad English indicated how difficult they found the task. n.s., *p* ≥ 0.05; *p < 0.05; **p < 0.01; ***p < 0.001. **c** Scatter plot showing the correlation between the responses to the questions ‘How difficult was the assignment?’ and ‘Is English your favorite subject?’ **d** Scatter plot showing the correlation between the responses to the questions ‘How difficult was the assignment?’ and ‘Is English a subject that you are good at?’. Larger bubbles correspond to more points. The approximately straight lines have intercepts of 0

When answering the question ‘How difficult was the assignment?’, many students chose answer options 1 (‘Very difficult’) or 2 (‘Difficult’) (Fig. 2). Regarding the questions ‘Is English your favorite subject?’ and ‘How difficult was the assignment?’, there was a significant difference in perceived difficulty level between students who answered ‘Yes’ versus ‘Definitely not’ or ‘No’ to the former question (Fig. 4a). Regarding the questions ‘Is English a subject that you are good at?’ and ‘How difficult was the assignment?’, there was no significant difference in perceived difficulty between students who answered ‘Neutral’, ‘Yes’, or ‘Very much so’ to the former question (Fig. 4a, b), but there was a significant difference between students who answered ‘Neutral’ versus ‘Definitely not’ or ‘No’ (*p* ≥ 0.05; *p < 0.05; **p < 0.01; ***p < 0.001, Fig. 4b). The correlation coefficient between the responses to the questions ‘How difficult was the assignment?’ and ‘Is English a subject that you are good at?’ was 0.5731 (*R*^2^ = 0.8365) (Fig. 4c), and that between ‘How difficult was the assignment?’ and ‘Is English your favorite subject?’ was 1.286 (*R*^2^ = 0.8121) (Fig. 4d).

**Fig. 5:**
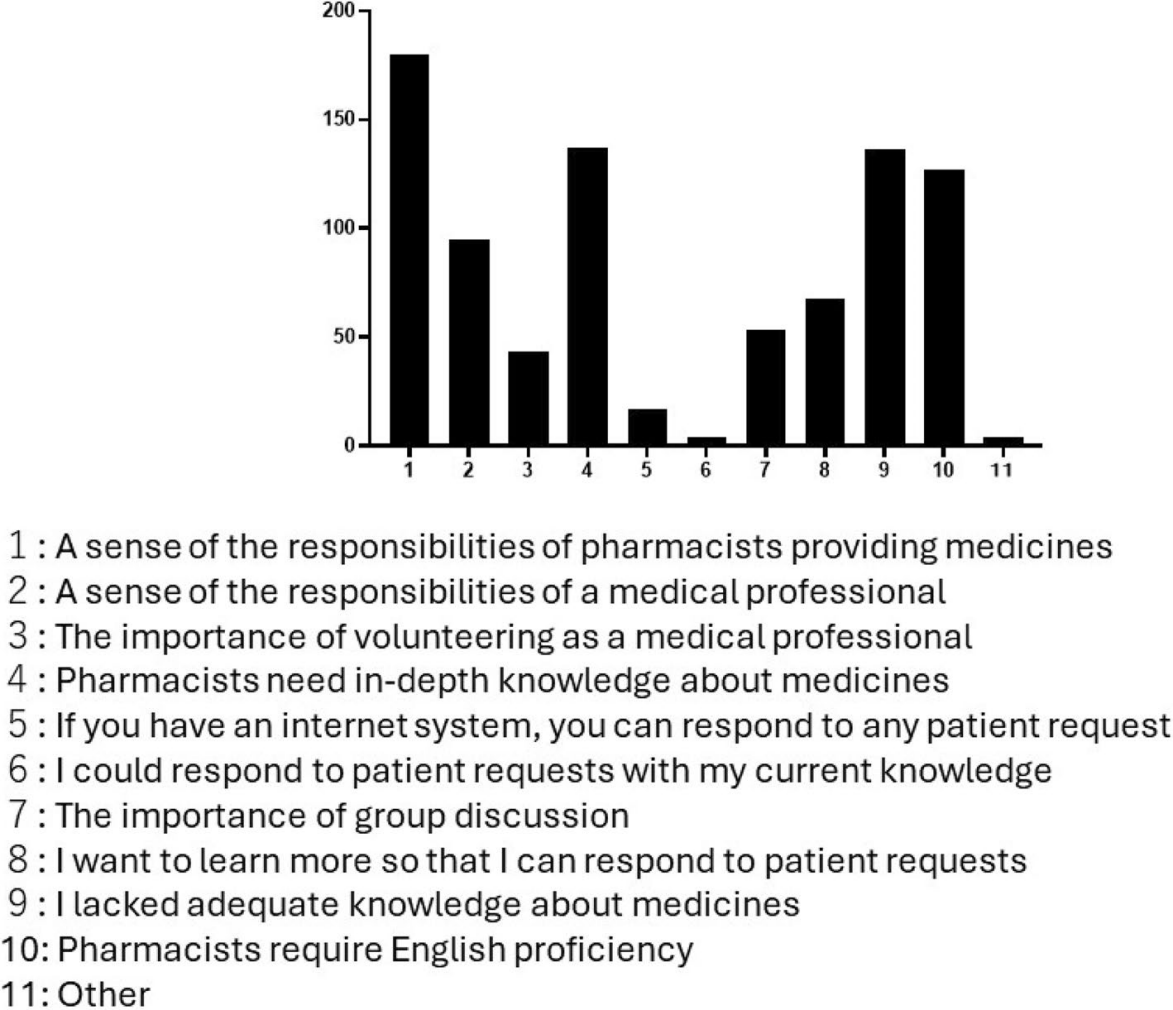
Distribution of responses to question 12 on the post-questionnaire

For question 12 of the post-questionnaire (‘What did you realize through the exercise?)’, the most common responses (in order) were ‘A sense of the responsibilities of pharmacists providing medicines’, ‘Pharmacists need in-depth knowledge of medicines’, ‘I lacked adequate knowledge on medicines’, and ‘Pharmacists must be proficient in English’ (Fig. 5).

### Qualitative analysis of ‘impressions of the exercise’

**Fig. 6:**
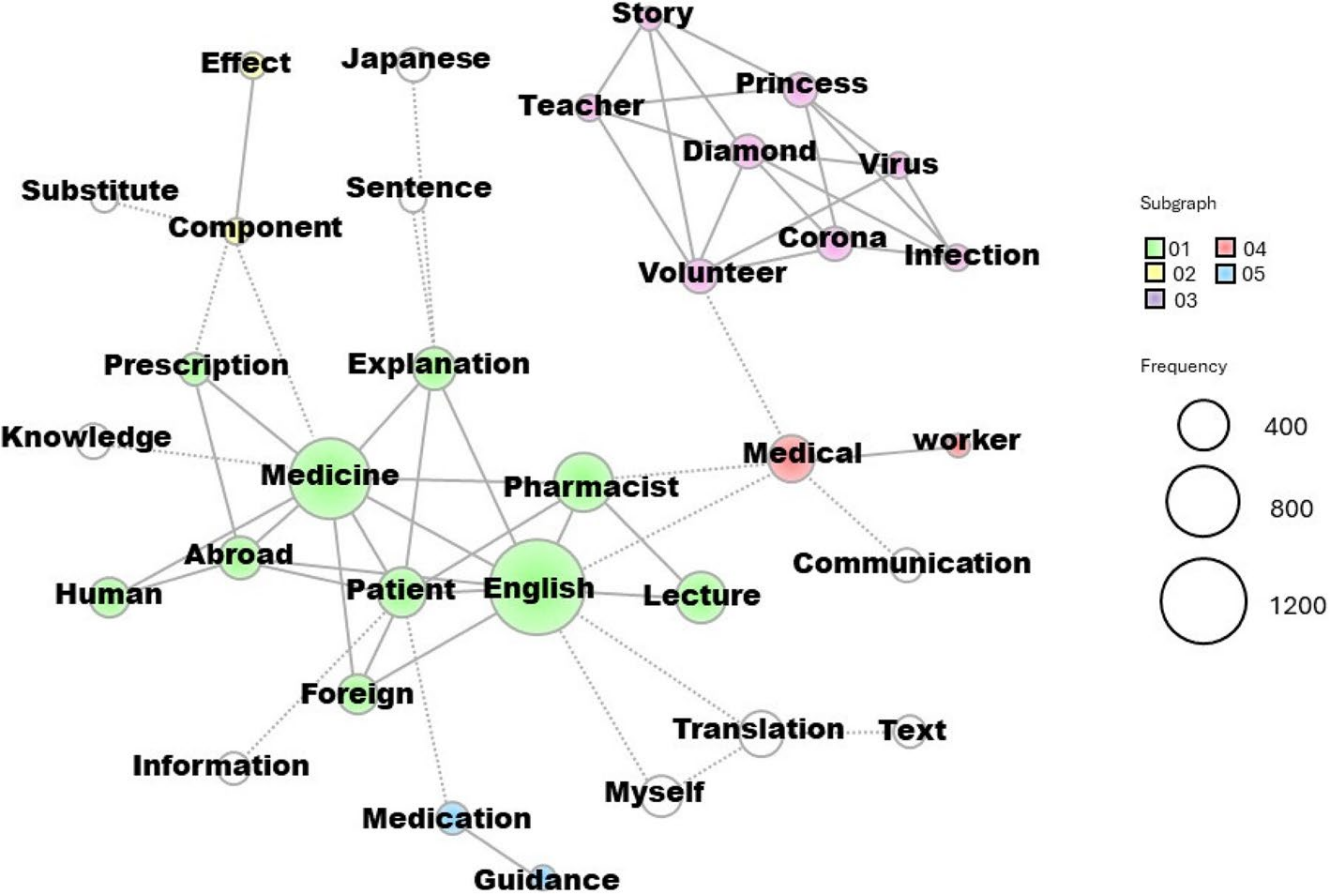
Co-occurrence network diagram derived via text mining. Frequently occurring words (nouns) and phrases are shown as large circles, and closely related words and phrases are connected with lines

In Subgraph 03 of the co-occurrence network diagram, ‘Diamond’, ‘Princess, ‘volunteer’, ‘teacher’, ‘story’, and other words are linked (Fig. 6). The opinions that were expressed in the ‘Impressions of the exercise’ homework assignment were as follows. ‘This time, the teacher told us about what actually happened on DP, and I was reminded once again of just how dire the situation was. I thought it was a very valuable experience to actually hear the story of that time’ (Student #45). ‘The exercise reflected the teacher’s actual experience, so it was very realistic and I learned a lot’ (Student #198). ‘I think this action reflects the fundamental desire of a medical worker to help people with medical care. I was very impressed with the determination’ (Student #94). It is thus clear that the author conveyed the reality of the experience by lecturing using photographs, and that students were able to perform their assignments with an awareness of the actual situation (128/291, 44.0%, Table 2).

Based on the following opinions expressed in the homework, it seems that we were able to convey the significance of supplying medical support during disasters as medical professionals (90/291, 30.9%, Table 2). ‘I learned that pharmacists play an active role in emergency situations related to infectious diseases, which were mentioned during the lecture, and also as medical support during disasters, and I wanted to be present in such a situation in the future’ (Student #121). ‘After listening to the lecture, I thought it would be a good idea to participate in such medical support in the future’(Student #204). ‘I think there are many people who are afraid to go there. Some people may go if the reward for participating is unimaginably high. I would like to know why you participated’ (Student #15).

Morphological analysis of the ‘Impressions of the exercise’ homework showed that the top 10 words included ‘English’, ‘medicine’, ‘pharmacist’, ‘lecture’, ‘patient’, and ‘explanation’ (Table 3). The co-occurrence network linkages are shown in Subgraph 01 (Fig. 6), which summarizes opinions on English; broadly speaking, there were two types of opinions. First, as shown in the comments that follow, many students realized that pharmacists need English skills (277/291, 95.2%, Table 2). ‘The practice of writing practical English sentences in the exercise was fun because it was something I had not really touched on in my previous English classes and studies’ (Student #43). ‘I did not really feel that English was necessary for pharmacists; this lecture was the first time I felt that it was so necessary’ (Student #201). ‘Even after completing the exercise, my dislike of English did not change much. But I was able to see that English can be useful, and I feel more positive about studying English than before’ (Student #272). ‘If a natural disaster such as a typhoon or earthquake occurs in a tourist destination, foreigners who do not understand Japanese may need medicine, so I realized that it is necessary to memorize English grammar and English vocabulary related to medical care’ (Student #198).

**Table 3:** The top 10 frequently occurring words of a results of morphological analysis about ‘impressions of the exercise’ assigned as homework

Rank	Words	Frequency
1	English	1570
2	medicine	1093
3	think	960
4	Pharmacist	576
5	necessary	468
6	This time	465
7	Japan	433
8	Lecture	395
9	Patient	392
10	feel	366

In the other type of comment (below), many students wrote that they understood the need to make their own decisions rather than relying solely on answers provided by AI features such as translation apps (115/291, 39.5%, Table 2). ‘I was using Google Translate for the assignments, but it sometimes makes mistakes, so I thought that I needed to understand English myself, and that I would be held responsible if I gave the wrong information to a patient’ (Student #115). ‘In the pre-questionnaire, I chose the option that English proficiency was not necessary as long as I had a translator, but after completing the assignments, I realized that English proficiency is absolutely necessary for pharmacists’ (Student #254). ‘Until now, I thought it was okay even if I wasn’t very good at English, but I realized that English is important for pharmacists. I thought I could handle it by using a translation application, but this exercise made me realize that I want to become a pharmacist who can understand the medicine myself and provide the medicine that the patient really wants’ (Student #202).

Regarding opinions related to ‘medicine’ and ‘knowledge’, linked in Subgraph 01 of the co-occurrence network, many students considered that, as pharmacists, they needed excellent drug knowledge, and their motivation to study drug treatment thus increased (67/291, 23.0%, Table 2). ‘There may be situations in which medical care needs to be provided in English during an emergency. In situations like this, I want to avoid delays in judgment and response due to a lack of English proficiency, and I thought that if I could speak English, I would be able to do more as a medical worker. I realized that it is necessary not only to fill one’s head with medical knowledge, but also to be able to respond flexibly to any situation in terms of language’ (Student #23). ‘I once again realized that pharmacists play an important role in medical care. I don’t have much knowledge yet, but I realized once again that I want to study every day in order to become a pharmacist who can be close to patients in the future’ (Student #108). ‘What made me think the most was the high level of clinical performance of pharmacists that would be required in the field. Through this lecture, I became more motivated to study English and other fields of pharmaceutical science’ (Student #266).

Based on the qualitative content analysis, three major themes emerged:

1) Importance of English proficiency, 2) Awareness of AI risks in clinical practice, and 3) Professionalism in pharmacy practice.

1. Importance of English proficiency: Many students expressed that the exercise heightened their awareness of the need for English proficiency in pharmacy practice. One student commented, ‘I realized how difficult it is to explain medication instructions in English. I need to study English more seriously’ (Student #243). Another student noted that they previously underestimated the necessity of English in pharmaceutical settings but now feel more motivated to improve their language skills.
2. Awareness of AI risks in clinical practice: Several students raised concerns about the limitations of AI-based translation tools. One student stated, ‘I initially trusted AI-generated translations, but after comparing them with human translations, I noticed critical errors in medication instructions’ (Student #119). Another reflected on the ethical responsibility of pharmacists to verify AI-generated content before relying on it in patient care.
3. Professionalism in pharmacy practice: Many students recognized the broader role of pharmacists in disaster response and patient communication. One reflection noted, ‘I had never considered that pharmacists could play a crucial role in emergency situations. This exercise made me think more about the responsibilities of my future profession’ (Student #69). Additionally, some students highlighted the psychological burden of making medication-related decisions under uncertain conditions.

These themes were not independent but interrelated. For instance, students who recognized the importance of English proficiency were also more likely to express concerns about over-reliance on AI, suggesting a potential link between language confidence and risk perception.

The plan is to further implement the new educational method and collect additional data to make learning even more effective.

The qualitative and quantitative data were consistent in illustrating key trends observed in student reflections. For example, the Wilcoxon signed-rank test showed a significant increase in students’ perception of the importance of English proficiency (Pre-Q3 vs. Post-Q4, *p* < 0.01). This statistical finding aligns with qualitative reflections, where multiple students expressed a heightened awareness of the necessity of English in pharmacy practice. One student stated, ‘I realized that my English proficiency was insufficient and that I need to study more seriously’(Student #68). Another commented, ‘Before this exercise, I didn’t think English was important for pharmacists, but now I understand its relevance’(Student #8).

Furthermore, the development of professionalism in pharmacy practice was evident in both thematic analysis and statistical results. Several students stated that they had a new understanding of the pharmacist’s responsibility to provide medicines in Q12 of the post-survey, which is consistent with an increased perception of professionalism. Representative statements included, ‘I never thought of pharmacists as important people in emergency medicine, but this exercise changed my perspective’(Student #83).

By integrating both statistical comparisons and qualitative insights, the findings maintain strong consistency with the data collected, ensuring that conclusions are directly supported by evidence.

## Discussion

Before the exercise, the author gave a lecture (using photographs) on the medical support offered during a disaster and his feelings at the time [16]. Analysis of the ‘Impressions of the exercise’ revealed that, after listening to the lecture, students were able to tackle their assignments with a sense of being in the field (128/291, 44.0%, Table 2).

The number of positive responses to the question ‘Would you like to study English more deeply if you had an opportunity?’ significantly increased after the exercise. This was also reflected in the ‘Impressions of the exercise’ homework assignment, where students stated said that they enjoyed learning English via actual cases, and that, because of the lecture, they realized that English was necessary. The fact that many such opinions were expressed shows that students feel the need to study English. However, there was no significant difference in the responses to the question ‘Do you want to be able to speak English?’ before and after the exercise, although there was a significant increase in the number of respondents who wrote ‘Pharmacists need to be able to read and write English rather than speak English’. The exercise conveyed the need to read and write in English; English communication skills are essential in clinical settings, and training programs that enhance student motivation to learn conversational English are required (277/291, 95.2%, Table 2) [23].

Regarding the question ‘Do you think you don’t need English skills if you have a translation application?’, there was a significant change in responses after the exercise. In the ‘Impressions of the exercise’ homework, many respondents stated that they accepted that pharmacists, who are medical experts, are ultimately responsible for any deficiencies in care caused by the use of AI (such as translation software). Thus, rather than relying on translation software, students felt the need to improve their English skills and make final decisions on their own (115/291, 39.5%, Table 2)

Many students answered the question ‘How difficult was the assignment?’ with ‘Very difficult’ or ‘Difficult’. As second-year students have not yet studied pharmacotherapy in depth, we explored whether the difficulty was related to poor English or a lack of pharmacotherapy knowledge. There were relationships of perceived task difficulty with ‘liking’ and proficiency in English.

We confirmed a relationship between the answers to ‘Is English a subject that you are good at?’ and ‘How difficult was the assignment?’ However, there was no significant difference in the perceived task difficulty among students who answered ‘Neutral’, ‘Yes’, and ‘5 Very much so’ to the question ‘Is English your favorite subject?’ (Fig. 4a), and the correlation coefficient between liking English and task difficulty was 0.5597 (*R*^2^ = 0.8284), indicating a correlation.

On the other hand, the answers to ‘Is English a subject that you are good at?’ were significantly different among students who answered ‘Neutral’, ‘Definitely not’, and ‘No’ to the question on perceived task difficulty (Fig. 4b). The correlation coefficient was 0.6315 (*R*^2^ = 0.8121) (Fig. 4d). Thus, a strong correlation was apparent between English proficiency and perceived task difficulty.

Although the task may have been difficult for second-year students who had not yet studied drug treatment in depth, the main problem was a lack of English skills; the perceived task difficulty was particularly strongly correlated with poor English proficiency and low English favorite.

Regarding question 12 of the post-questionnaire, ‘What did you realize through the exercise?’, most students chose the answer option ‘A sense of the responsibilities of pharmacists providing medicines’. Thus, through the exercise, we conveyed that pharmacists must take responsibility for providing medicines. The next most common responses were ‘Pharmacists need in-depth knowledge about medicines’ and ‘I lacked adequate knowledge about medicines’. In addition, in the ‘Impressions of the exercise’ homework, many students stated that the exercise prompted a strong desire to improve their clinical ability, and their motivation for clinical learning increased. Thus, by conducting exercises based on a real emergency, students not only increased their motivation to learn English but also prioritized professional education in pharmaceutical sciences, which was about to begin in earnest in year 3 of their studies.

After learning the real situation in the field based on the author’s experience, as described in the lecture, students tackled their assignments. They learnt many things that are difficult to teach via lectures alone, such as the dangers of using AI technology in clinical settings and the mindset of medical professionals. However, a single report (such as this paper) cannot fully evaluate the effects of learning in various fields (including English), the mental attitudes of medical professionals, and good drug therapy practice.

## Conclusion

This paper presents a detailed analysis of the unique teaching method used in actual undergraduate education and its educational effectiveness by using the challenges encountered by pharmacists who provided medical support on the cruise ship Diamond Princess during the COVID-19 outbreak. By using this disaster-based task, we were able to not only students’ motivation to both learn English and receive professional education in the pharmaceutical sciences increased, but also learn about the dangers arising when using AI technology in clinical settings and the mindset of medical professionals. This is the first report on the medical support activities of pharmacists on the Diamond Princess cruise ship, and the first report to use a real-life example of pharmacists’ activities during a disaster in actual education.

## Data Availability

The datasets used and/or analysed during the current study are available from the corresponding author on reasonable request.
